# Acute Nephritis Complicating Infectious Diseases

**Published:** 1918

**Authors:** J. D. Burt


### ACUTE NEPHRITIS COMPLICATING INFECTIOUS DISEASES.

**By J. D. Burt, M.D.**

Toxic infectious diseases most certainly occupy first place in the pathogenesis of nephritis. Prominent among the numerous infectious diseases calculated to irritate and inflame the kidneys may well be mentioned: Scarlet fever, syphilis, general acute eczema, erysipelas, typhoid fever, grippe, measles, mumps, etc. The diseases in which the kidneys present the pathognomonic microbes in the arteries or the glomeruli are anthrax, pneumônia, tuberculosis, strepto and staphylococci infections. In such cases both the germ and the toxins are present. The presence of germs in the kidney tissue tend to traumatism and congestion. The toxins of the germs no doubt are of greater concern and do far greater damage to the kidney, acting especially in the epithelium.

Tuberculin injected for diagnostic purposes is quite likely to cause symptoms of nephritis with kidney changes. Cases of acute infections wiht intense intoxication induce inflammatory degenerative changes with the epithelia, interestitial and vascular lesions. These facts present conclusive proof that the kidneys are affected by infectious germs and their toxins in the same manner that they are affected by mineral poisons.

From a practical standpoint, it matters not whether the poison acts directly on the gidney epithelium, the uriniferous tubes or the arterioles resulting in arterio-scerosis, or whether the lesion may be associated, degenerative or inflammatory. As a clinical fact, we know that nephritis is induced by acute infectious diseases, both of unknown microbic origins, as in scarlet fever, measles, smallpox, etc., and also from the list of known origin infectious diseases, as grippe, pneumonia, typhoid fever, tetanus, erysipelas, puerperal fever, etc.

It is important to realize that these various infections do not attack the kidney with the same violence nor with the same tenacity. Those suffering from scarlet fever and syphilis give the worst form of kidney lesions, while many cases of other types may be mild in degree, presenting only a transitory albuminuria as the leading symptom with apparently readily and rapid elimination. It must be understood, however, that any case of nephritis so long as any sort of irregular symptoms remain is a case of gravity and although primarily slight may become permanently grave and result in a well-defined chronic nephritis with all its heart lesions and other bad complications.

Edema of the bronchial tubes and lungs must not be misundehstood, if it is dependent on nephritis for its lesions. Pleural effusions as a result of nephritis must not be regarded as pleurisy. It is well to remember that albuminuria is always prseent in acute nephritis, while it is not necessarily so in cases of the chronic type. The prognosis in these cases depend upon the infection that produces the disease and the ability of the physician to recognize the case and put the patient on correct treatment. As a rule, this class of cases is very amenable to treatment. The diagnosis of acute nephritis is made in the laboratories, but there are well-defined symptoms which usually accompany all cases, such as headache, backache, trouble with the vision, muscular nephritis. In fact, every patient who has had scarlet fever should have his urine tested every week for at least two or three months. The other infectious diseases do not have such a bad effect on the kidneys as scarlet fever, but the urine should be examined a few times, and if there is any symptoms suggestive of nephritis they should be closely watched and if laboratory tests stage that the disease is most amenable to treatment. First symptoms are in children following scarlet fever usually edema about the face and eyes, which develops casually in the course of a convalescence and as a rule without fever. In older people, uremia may be the first symptom. Sometimes the disease is not detected only by frequent analysis of the urine.

Knowing how to interpret symptoms and make an early diagnosis in these diseases, and keeping the patient on correct diet and treatment, is the only way one will be able to cut the mortality below the 33 per cent. it now has, and prevent the disease from going on into subacute and chronic nephritis with all its complications.

The treatment of acute nephritis requires immediate bed rest in a warm but properly ventilated room. Our aim from the first is to prevent heart lesions and other complications and to prevent further kidney irritations. The diet should consist of milk and water; all synthetic drugs and dieuretics should be avoided. If there is fever, cold sponges should be used with great care; warm water will usually be as effective as the cold, although the patient should have plenty of cool air to breathe. Hot baths and packs to the back will have a tendency to relieve the congestion of the kidneys by increasing the secretion of the skin. Milk and water is the proper diet when albumin first appears in the urine, and should be rigidly enforced, but if the patient's stomach rebels at the milk, one can give thin gruel, oatmeal, malted milk or cereal milk. Barley water has some nourishment and can be given freely; a very refreshing drink is made from a teaspoonful of cream of tartar in a pint of boiling water, to which is added the juice of a lemon or an orange; may add a little sugar. This acid is burned into an alkali in passing through the system. If the patient's stomach is much disturbed and vomits frequently, it is best to withhold all foods for awhile and give liberal quantities of hot water; however, one cannot continue this treatment long, for it tends to acidemia, which would add its toxins to those already present and tends to produce uremia.

Bismuth subcarbonate, fifteen grains every two or three hours, is usually sufficient to control the vomiting. To produce elimination the body should be kept warm, hot sponge baths should be given; medicines and hot packs should not be given to produce sweating; nothing is indicated but warm drinks and warm applications to the back. The intestinal canal should be cleaned out by a calomel purge, but too free purgation is not indicated Daily doses of salts or oil should be given if needed to give two or three operations from the bowels daily. If the heart is weak, one should be very careful in giving purges. Dieuretics should not be given except pure water, to which may be added citrate of potash or soda in fruit juices, such as orange or lemon. If the patient is restless and cannot sleep, chloral hydrate and bromide of potash can be given. If the heart is weak, morphine could be substituted for the chloral, though as a rule one should not give an opiate in this disease. Synthetic drugs and coal-tar derivatives are contraindicated on account of the bad effect on the kidneys.

Our aim in the treatment of acute nephritis complicating infectious diseases as stated is to rid the blood of the germ and their toxins of the disease, and this is done by keeping the skin active. Too profuse sweating weakens the patient and does not get rid of the toxins in the blood, but a gentle action of the skin must be maintained by hot drinks, electric pads to the back, etc. If there is high blood pressure with threatened uremia, blood-letting is sometimes a life-saving operation. An acute nephritis complicating infectious disease runs its course in two or three weeks to two or three months. During the convalescence stage the patient can have a more liberal diet; he can have bread, butter and vegetables, but meat should be very cautiously given. Tincture of iron three times a day should be given and the patient kept from the cold; the body should be kept warm, and warm baths taken every day. If it is winter, and the patient can afford it, should be sent to a warm climate.

To recapitulate, acute nephritis following infectious disease is more amenable to treatment when the first symptoms are recognized and the patient placed on correct diet and care, also that one should never give up hope, for some will get well after having appeared to have been in a moribund condition.

In closing, I will say that if this will cause one doctor to be more on the lookout for kidney lesions when he has a case of scarlet fever or other infectious disease, I will feel that I have been well paid for this feeble attempt to write.—Texas Medical Journal.

### STEAROSAN: SANTAL OIL IN IMPROVED FORM.

After a comparatively short course of treatment with santal oil the stomach sometimes rebels, and the patien t suffers from such symptoms as gastric distress, nausea, disagreeable eructations and loss of appetite. This necessitates a discontinuance of the medicament and means an annoyance to the physician and a loss to thepatient. It was to obviate this——to secure the therapeutic virture of oil of santal without its deleterious effects——that Stearosan was evolved.

Stearosan is santalyl steartte——santalol combined withh stearic acid——a true chemical compound, equivalent to about 50 per cent of santal oil. It is supplied by Parke, Davis & Co. in 10—minim elastic gelatin globules, in boxes of 25 and 100. The average dose is one globule immedately after each meal, to be increased to two or three as indicated.

A complimentary package of Stearosan will be sent to any dphysician who wishes to teset the therapeutic efficacy of the product. Requests should be addressed to Parke, Davis & Co. at their home offices and laboratories, Detroit, Mich.

